# Sensitivity analysis of physics and planning SmartArc parameters for single and partial arc VMAT planning

**DOI:** 10.1120/jacmp.v13i6.3760

**Published:** 2012-11-08

**Authors:** Kai Yang, Di Yan, Neelam Tyagi

**Affiliations:** ^1^ Department of Radiation Oncology William Beaumont Hospital Royal Oak MI USA

**Keywords:** SmartArc, leaf speed, control points, VMAT

## Abstract

We investigate the sensitivity of various physics and planning SmartArc parameters to generate single and partial arc VMAT plans with equivalent or better plan quality as IMRT. Patients previously treated with step‐and‐shoot IMRT for several treatment sites were replanned using SmartArc. These treatment sites included head and neck, prostate, lung, and spine. Effect of various physics and planning SmartArc parameters, such as continuous vs. binned dose rate, dynamic leaf gap, leaf speed, maximum delivery time, number of arcs, and control point spacing, were investigated for Elekta Axesse and Synergy linacs. Absolute dose distribution was measured by using the ArcCHECK 3D cylindrical diode array. For all cases investigated, plan metrics such as conformity indices and dose homogeneity indices increased, while plan QA decreased with increasing leaf speed. Leaf speed had a significant impact on the segment size for low dose per fractionation cases. Constraining leaf motion to a lower speed not only avoids tiny large leaf travel and low‐dose rate value, but also achieves better PTV coverage (defined as the volume receiving prescription dose) with less total MUs. Maximum delivery time, the number of arcs, and the spacing of control points all had similar effects as the leaf motion constraint on dose rate and segment size. The maximum delivery time had a significant effect on the optimization, acting as a hard constraint. Increasing the control point spacing from 2 to 6 degrees increased the PTV coverage, but reduced the absolute dose gamma passing rate. Plans generated using continuous and binned dose rate modes did not show any difference in the quality and the delivery for the Elekta machines. Dosimetric analysis with a 3D cylindrical QA phantom resulted in 93.6%–99.3% of detectors with a gamma index 3%/2 mm <1 for all cases.

PACS number: 80

## I. INTRODUCTION

Volumetric‐modulated arc therapy (VMAT) is a truly dynamic rotation delivery technique where MLC shapes, leaf motion, gantry speed, and collimator rotation are changing constantly during beam on. VMAT has potential benefits compared to IMRT in terms of increased tumor control and reduced toxicity to normal tissue.^1^, ^3^This is because VMAT delivers dose to the target from multiple angles instead of limited angles, resulting in more degrees of freedom for the optimizer to reach an optimal solution. However, the quality of VMAT plans is highly dependent on the implementation of VMAT and the optimization algorithm used. It may vary, depending on the treatment planning system (TPS) used.^4^, ^7^

SmartArc is the VMAT algorithm in ${\text{Pinnacle}}^{3}$ 9.0 treatment planning system (Philips Radiation Oncology Systems, Fitchburg, WI) for clinical use. A description of the SmartArc optimization algorithm was published by Bzdusek et al.^(4)^ and the initial dosimetric evaluation was performed by Feygelman et al.^(8)^ These studies, however, were limited to evaluating the dosimetric effect of control point spacing and maximum delivery time on plan quality. In addition to the control point spacing, there are various other options in SmartArc user‐selectable and physics parameters. Their optimal range needs to be understood clearly for efficient clinical planning. For this purpose, we systematically performed a sensitivity analysis of various important SmartArc physics and planning parameters to generate efficient single and partial arc VMAT plans. One objective of this study was to investigate whether SmartArc was capable of generating efficient single and partial arc VMAT plans in terms of dosimetric quality and delivery efficiency. The other objective was to create a list of optimal parameters that can serve as guidance for efficient clinical planning for various treatment sites.

## II. MATERIALS AND METHODS

### A. Physics and planning parameters

SmartArc algorithm uses Pinnacle's existing machine parameter optimization, where all dynamic constraints (such as leaf speed, gantry speed, and dose rate) are taken into account during optimization. The SmartArc physics parameters that we investigated were: dynamic leaf gap and continuous vs. binned dose rate. The user‐selectable planning parameters investigated are leaf speed, maximum delivery time, control point spacing, arc length, and number of arcs.

#### A.1 Leaf speed

Leaf speed is defined in terms of leaf travel distance (in cm) per gantry angle (in degrees). In constraint leaf motion (cm/deg) option, the user specifies the maximum leaf travel distance that is allowed between adjacent control points. For a leaf motion constraint set to 0.5 cm/deg, and the control point spacing set to 4 deg, the leaf travel distance between adjacent control points will be limited to 0.5×4=2 cm. The leaf speed is directly related to the amount of modulation allowed in the algorithm to generate the plan. A larger leaf speed enables the leaf to travel a wider range between control points, which results in a higher modulation in the plan with lower dose rate values. The optimization menu allows user to choose this option under the leaf motion constraint tab. The recommended default leaf speed is 0.46 cm/deg. We looked at the effect of the following leaf speeds on plan quality: 0.12, 0.18, 0.25, 0.46 (default leaf speed), and 0.8 cm/deg.

#### A.2 Maximum delivery time

Maximum delivery time is the maximum time allowed for the plan delivery defined by the user. If a machine is able to complete a 360 degree rotation while moving at a maximum gantry speed of 6 degree/s, the minimum treatment time would be 60 s. However, the gantry may need to move slower during delivery to allow some leaves to travel to their correct positions from one control point to the next, and to allow sufficient variations in dose rate between control points, as well. The maximum delivery time can take any value as the input. Our starting point was the time for an ideal delivery based on the maximum dose rate and dose/fraction for the plan. The maximum treatment time was varied around the optimal time and its effect was evaluated on various plan metrics.

#### A.3 Number of arcs, arc length, and control point spacing

Multiple arcs can be generated with the same or different settings in each of the individual arcs. SmartArc supports two ways to create multiple arcs. Users can either specify settings for each of the individual arcs by creating separate beams for separate arcs, or use the dual arcs option. Dual arcs only allows users to assign the first arc and the algorithm will use the same setting to generate the second arc with opposite rotation. We investigated the effect of both scenarios — multiple arcs from single arc or defining multiple arcs in the beginning. We also studied the effect of control point spacing (6, 4, 3, and 2 degrees) on single or partial arc plans. Majority of the plans were generated with 4 or 6 degree control point spacing to reduce the planning time. The practice at our institution is to plan with 4 or 6 degree control point spacing and then use an interpolation script to evaluate the plan at 2 degrees. If there is a significant difference in the plan, the plan is reoptimized using the smaller control point spacing. The 2 degree plan is used for all QA evaluations.

#### A.4 Continuous vs. binned dose rate

The SmartArc optimizer performs in four different modes depending on how the machine is commissioned. Varian linacs operate in continuous dose rate mode, whereas Elekta linacs operate in discrete dose rate mode. For the binned dose rate mode, SmartArc does not do the binning until it gets close to the end of the optimization. A few iterations from the end, it takes the appropriate dose rates, which have been varying throughout the optimization within the range of minimum and maximum values, and stratifies them into their closest bin value. In continuous dose rate mode, the optimizer has the most freedom to find a solution. Although our institution has Elekta linacs, we generated plans in both continuous and binned dose rate mode to look at the effect on planning, delivery, and QA for various disease sites. These dose rate modes were specified in the physics module of Pinnacle TPS. We allowed the continuous dose rate to vary between 100 to 600 MU/min. Binned dose rate values were specified as: 500, 460, 228 and 114 MU/min for a 6 MV photon beam.

#### A.5 Dynamic leaf gap

In addition to static leaf gap specified in physics modeling for IMRT planning, a new parameter, called dynamic leaf gap, also needs to be specified for dynamic arcs. First, the optimizer decides the maximum number of leaves that can be used to define an aperture for any control point. Leaf pairs that remain closed during the entire beam delivery are positioned under the jaws with the static leaf gap. Of the remaining leaf pairs, some may be closed at certain control points, but open at others. These leaf pairs are positioned with the new dynamic leaf gap when they are not used at a control point in the field. The dynamic leaf gap is necessary to avoid any collision and to minimize the time required to park those leaves. It has to be greater than or equal to the static leaf gap. We investigated the effect of using a 1, 2, and 3 mm dynamic leaf gap.

### B. Treatment planning and delivery

A number of patients previously treated with step‐and‐shoot IMRT for various treatment sites were replanned using single and partial arcs. These treatment sites include head and neck (2 Gy x 35 fx), prostate (3.2 Gy x 20 fx, 1.8 Gy x 43 fx), lung (12 Gy x 4 fx), and spine (18 Gy x 1 fx). The majority of the plans were delivered on Elekta Axesse linac (Elekta, Stockholm, Sweden) equipped with a beam modulator using 6 MV photon beam, except the head‐and‐neck case that was delivered on the Elekta Synergy linac. Treatment plans were transferred via DICOM RT (DICOM, Rosslyn, VA) from the TPS to the MOSAIQ (v. 1.6) record and verify (R&V) system (IMPAC Medical Systems, Sunnyvale, CA). Most of the plans in this study were created with 4 or 6 degree spacing. The physics parameters were set to deliver continuous dose rate for all these plans. Plan quality was assessed by calculating the radiation conformity index (RCI) and dose homogeneity index (DHI) defined as:^(9)^

(1) $$\begin{array}{rl} & RCI=\frac{V_{D_{p},PTV}}{V_{D_{p},Body}};\\ & DHI=\frac{D_{99.5\%}}{D_{0.5\%}} \end{array}$$

where $V_{Dp,PTV}$ and $V_{Dp,Body}$ are the volume within the PTV and the body that receives the prescription dose or higher.

### C. Dosimetric QA

Absolute dose distribution was measured using the ArcCHECK cylindrical diode array (Sun Nuclear Corporation, Melbourne, FL). This device has been previously verified for IMRT, Helical Tomotherapy, and VMAT dosimetry.^10^, ^11^ A gamma analysis criteria of 3% absolute dose error and 2 mm distance to agreement (DTA) was used to compare the measured and calculated dose.^(12)^ Detectors receiving at least 4% of the prescription dose were included in the analysis. For QA calculations, the plans were interpolated to 2 degree control point spacing.

## III. RESULTS

### A. Planning parameters

#### A.1 Leaf speed

**Figure 1:**
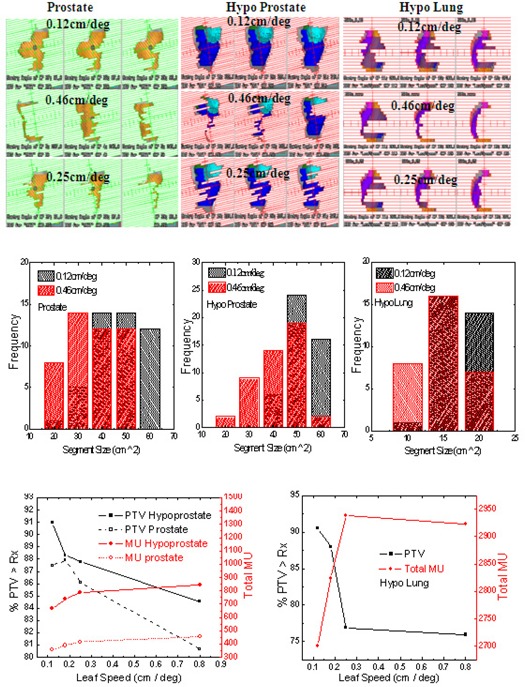
Comparison of control points (a) with constraint leaf motion option set to 0.12 cm/deg, 0.46 cm/deg, and 0.25 cm/deg; comparison of segment size distribution (b) for leaf motion options set to 0.12 cm/deg and 0.4 cm/deg; influence of varying leaf speed (c) on total MU and PTV coverage for prostate, hypoprostate, and hypolung SmartArc plans.

**Figure 2:**
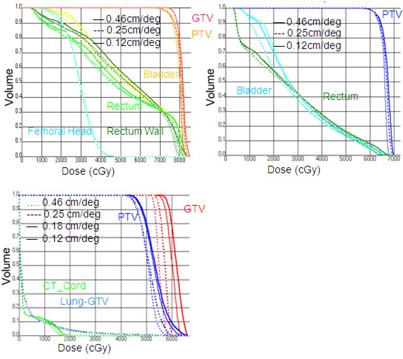
Comparison of DVHs of prostate (top left), hypoprostate (top right), and hypolung (bottom left) SmartArc plans with constraint leaf motion option set to 0.12cm/deg (thick solid line), 0.46 cm/deg (thin solid line), and 0.25 cm/deg (thick dashed).

Leaf speed shows sensitivity to segment shapes depending on the dose/fraction as shown in (Fig. 1a) for prostate and hypofractionated prostate and lung cases. These patients were planned using 6 degree control point spacing on Elekta Axesse machine with beam modulator. Three adjacent control points have been shown with varying leaf motion constraint. The segment shape starts to become complex at 0.25 cm/deg for prostate cases and at 0.18 cm/deg for hypofractionated prostate (or hypoprostate) cases. For prostate cases, without constraining the leaf motion, almost one‐third of the leaf segments were complex. This is also evident from the segment size distribution shown in (Fig. 1b) for prostate and hypofractionated lung (or hypolung) plans with 0.12 and 0.46 cm/deg leaf motion constraint. The frequency of larger shape segments is much higher for constrained leaf position, as compared to unconstrained leaf position for all three cases investigated. The segment areas, when constraining the leaf motion, were $41.0\pm 9.2\;{\text{cm}}^{2}$, $46.6\pm 7.1\;{\text{cm}}^{2}$, and $14.1\pm 2.2\;{\text{cm}}^{2}$ for prostate, hypoprostate, and hypolung case, respectively. The corresponding segment areas without constraining the leaf speed were $31.7\pm 11.0\;{\text{cm}}^{2}$, $36.5\pm 9.4\;{\text{cm}}^{2}$, and $12.4\pm 3.6\;{\text{cm}}^{2}$, respectively.

(Figure 1c) shows the PTV coverage and total MU as a function of the leaf speed for hypoprostate, prostate, and hypolung SmartArc plans. For all cases, the MU decreased and the PTV coverage increased by constraining the leaf motion to lower speed. Specifically, by constraining the leaf motion to lower speed, the PTV coverage improved by 8% for prostate, 5% for hypoprostate, and 18.7% for the hypolung plan while, at the same time, the total MU reduced by 22.2% for prostate, 23.5% for hypoprostate, and 8.1% for the hypolung plan. The DVHs in Fig. 2 show the coverage for target and normal structure for different leaf speeds. The effect on normal structures was not very significant.

Table 1 shows the effect of varying leaf speed on delivered treatment times, MUs, plan metrics, and plan QA performed using ArcCHECK cylindrical phantom for various sites. The delivery time and MUs go up significantly for plans with high leaf speed constraint due to more frequent beam hold‐offs caused by complex segments resulting in a rapid MLC movement during delivery. An increasing trend was also seen for the plan metrics RCI and DHI. The absolute dose gamma passing rate decreased as the leaf speed was increased. This is most significant for the hypoprostate and SRS spine plans where the passing rate dropped from 96% to 85% and 90% to 78%, respectively, implying that the leaf speeds are changing very rapidly for the higher leaf speeds plan. The change in passing rate was negligible for the hypolung plan. Interpolated QA plan result obtained using the 2 degree interpolation script is also shown, along with the original QA plan result.

**Table 1:** Effect of varying leaf speed on treatment time, total MUs, plan quality metrics such as conformity index (RCI) and dose homogeneity index (DHI), and plan QA using ArcCHECK 3D cylindrical phantom. Interp. Plan refers to QA plans interpolated to 2 degree control point spacing.

			*Maximum Delivery Time (s)*					*ArcCHECK (3%/2 mm)*	
*TPS Parameter*	*Treatment Site*	*TPS Parameters Values*	*TPS*	*Delivery*	*Total MU*	*RCI*	*DHI*	*Ori. Plan*	*Interp. Plan*
	Prostate	IMRT	/		542.2	0.947	0.774		/
		0.12	90	102.1	353.8	0.984	0.926	92.8	95.7
		0.18	90	111.8	388.6	0.981	0.912	90.2	93.7
		0.25	90	124.8	417	0.983	0.818	92.5	94.9
		0.46	90	168.2	457.2	0.990	0.844	89.7	91
		0.8	90	170	455.2	0.991	0.847	87.2	88.6
	Hypoprostate	IMRT	/		893.7	0.842	0.907		/
		0.12	90	108	664.9	0.793	0.842	96.7	99.3
		0.18	90	110.8	736.6	0.811	0.860	95.2	98.5
Leaf Speed		0.25	90	121.6	789.3	0.797	0.865	94.7	97.3
Constrain		0.46	90	165.4	842.9	0.832	0.878	88.3	91.9
(cm/deg)		0.8	90	162.2	842.6	0.847	0.875	85.2	90.4
	Hypolung	IMRT	/		2225.1	0.799	0.706		/
		0.12	300	356.4	2700.7	0.912	0.679	92.8	93.5
		0.18	300	376.9	2824.1	0.911	0.700	92.4	94
		0.25	300	395	2938.6	0.958	0.712	93.4	95.8
		0.46	300	394.4	2923.1	0.977	0.746	92.5	95.4
		0.8	300	394.1	2923.1	0.976	0.746	92.3	95.6
	SRS Spine	IMRT	/		7330	0.918	0.590		/
	(T12‐L3)	0.18	660	582	4077.5	0.873	0.426	90.5	96.4
		0.25	660	609.4	4286.7	0.908	0.456	85.6	94.9
		0.6	660	643.7	4512.0	0.924	0.491	78.1	81.5

#### A.2 Maximum delivery time

**Figure 3:**
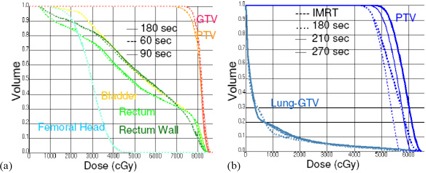
Comparison of DVHs of prostate SmartArc plans (a) with maximum delivery time at 60 (thick dashed), 90 (thick solid), and 180 (thin solid) seconds. Leaf motion constraint was set to 0.12 cm/deg for all plans. Comparison of DVHs of hypolung SmartArc plans (b) with maximum delivery time at 180 (thin dashed line), 210 (thin solid line), 270 s (thick solid line), and IMRT (thick dashed line).

(Figures 3a) and (b) show DVHs of prostate and hypolung SmartArc plans with varying maximum delivery time in planning. The PTV coverage and the OARs sparing remained the same as the maximum delivery time varied from 60 s to 180 s for the prostate plan. For hypolung plan with low maximum delivery time settings, the PTV coverage was comparable with the IMRT plan, but improved significantly when the maximum delivery time increased. Table 2 shows the effect of maximum delivery time on the dose rate distribution for the same prostate and hypolung SmartArc plans. As the maximum delivery time is increased for prostate, the optimizer varies the dose rate quite rapidly as compared to the lower time. However, the effect was quite opposite for hypolung plans.

**Table 2:** Effect of maximum delivery time on dose rate for prostate plan (top) and hypolung (bottom) SmartArc plan (control point spacing=6deg, leaf speed=0.12 cm/deg).

*Maximum Delivery*	*Control Points (Prostate with 45 control points)*	
*Time (s)*	>300 (MU/min)	>450 (MU/min)
180	12	7
90	12	6
60	25	17
*Maximum Delivery*	*Control Points (Hypolung with 30 control points)*	
*Time (s)*	>450 (MU/min)	>550 (MU/min)
180	30	30
210	29	27
240	23	23
270	21	18

#### A.3 Control point spacing, arc length and number of arcs

##### A.3.1 Number of arcs

**Figure 4:**
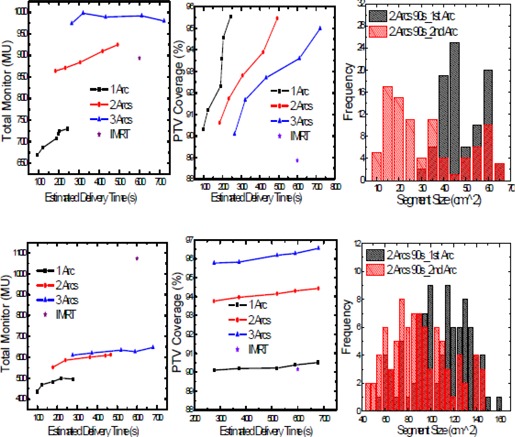
Influence of number of arcs on total MU, PTV coverage, and segment size distribution for hypoprostate (top) and HN (bottom) SmartArc plans.

Figure 4 shows the effect of number of arcs on total MU, PTV coverage, and segment shape distribution for hypoprostate (top graphs) and head and neck (bottom graphs) SmartArc plans. Different data points on the graphs show the plans that were obtained using different maximum delivery times. The segment size distribution for the two‐arc plan corresponds to 90 s maximum delivery time for each arc. Single arc can achieve similar PTV coverage as IMRT using 30% less MUs for the hypoprostate plan and 50% less MUs for the head‐and‐neck plan. For both plans, MU increased with the number of arcs, although the effect was more significant from single arc to dual arc than for dual to three arcs. The frequency distribution for segment shapes shows much larger segment areas for the first arc as compared to the second arc.

##### A.3.2 Control point spacing

Increasing the control point spacing from 2 to 6 degrees increased the PTV coverage and reduced the total monitor units, as shown in Table 3, for prostate, hypoprostate, and hypolung cases planned without constraining the leaf speed while keeping other parameters constant. The PTV coverage increased by 2%, 1.6%, and 12% for the prostate, hypoprostate, and hypolung cases, respectively, as the control point spacing increased from 2 to 6 degrees. The corresponding decrease in MUs for these cases was 6.8%, 2.1%, and 4.1%. The absolute dose gamma passing rate, however, decreased by 3.9%, 6.9%, and 1.7% for the prostate, hypoprostate, and hypolung cases, respectively, as the control point spacing increased from 2 to 6 degrees. Interpolated QA plan results obtained using the 2 degree interpolation script shows passing results that are similar to the 2 degree plans.

**Table 3:** Effect of control points spacing on total MUs, PTV coverage, and plan QA for prostate, hypoprostate, and hypolung SmartArc plan (no leaf speed constraint). Interp. Plan refers to QA plans interpolated to 2 degree control point spacing.

					*ArcCheck (3%/2 mm)*	
*Treatment Site*	*CP Spacing (deg)*	*Maximum Delivery Time (s)*	*Total MU*	*% PTV coverage*	*Ori. Plan*	*Interp. Plan*
Prostate	2	90	478	90.6	93.8	/
	4	90	465.2	91.6	92.4	94.2
	6	90	447.5	92.6	89.9	92.6
Hypoprostate	2	90	872.8	90.7	95.7	/
	4	90	858.5	91.5	93.8	95.1
	6	90	855.2	92.2	88.8	92.9
Hypolung	2	300	2810.6	78.4	94.4	/
	4	300	2803.5	86.2	94.0	94.7
	6	300	2700.7	90.5	92.7	93.2

#### A.4 Continuous vs. binned dose rate

Plans generated using continuous and binned dose rate modes did not show any difference in plan quality and delivery for the Elekta machines. By allowing the dose rate to be continuously variable, the optimizer has the most freedom to find a solution during most of the optimization. Table 4 shows the comparison of total MUs, PTV coverage, and plan QA for prostate, hypoprostate, and hypolung cases planned using binned and continuous dose rates. We found that continuous dose rate mode works very well with Elekta machines that have binned dose rate mode. This is because the Elekta machine's servo controls the gantry rate to achieve variable MU per degree, even with binned MU per minute. The absolute dose QA passing rate for the continuous dose rate plans is within 1%, compared to that of similar quality binned dose rate plans, and is always greater than 92.6% if a cylindrical QA device is being used.

**Table 4:** Comparison of total MUs, PTV coverage, and plan QA for prostate, hypoprostate, and hypolung SmartArc plan generated using binned and continuous dose rate mode (control point spacing=6deg, no leaf speed constraint). Interp. Plan refers to QA plans interpolated to 2 degree control point spacing.

					*ArcCheck*	
*Treatment Site*	*Dose Rate*	*Maximum Delivery Time (s)*	*Total MU*	*% PTV coverage*	*Ori. Plan*	*Interp. Plan*
Prostate	Binned	90	453	93.3	89.3	91.5
	Continuous	90	447.5	92.6	89.9	92.6
Hypoprostate	Binned	90	822.3	92.3	88.5	92.3
	Continuous	90	855.2	92.2	88.8	92.9
Hypolung	Binned	300	2808.5	95.1	92.4	94.3
	Continuous	300	2858	95.1	92.5	94.6

#### A.5 Dynamic leaf gap

Dynamic leaf gap did not show any effect on dosimetric coverage. However, the plans were undeliverable below 1.5 mm dynamic leaf gap. Even at 2 mm leaf gap some of the plans were undeliverable. Three millimeters (3 mm) was the optimal dynamic leaf gap for both the Elekta Axesse and Synergy machines.

## IV. DISCUSSION

### A. Planning parameters

#### A.1 Leaf speed

Tiny separated segment shape shows up in both prostate cases without leaf motion constraint because leaves can travel a long distance into the field with these higher speeds. These tiny segment control points are not favorable in delivery and fewer MUs are typically assigned to these control point. This means the leaf travel time could be longer than the time to deliver the MU for that control point, in which case the dose rate drops significantly for leaves to catch up during delivery. This was most noticeable during delivery of low MU control point with a high‐dose rate as in prostate cases, where typical MUs per control point was 20.

Leaf speed did not have much effect on the OARs, as shown in Fig. 2. For prostate cases, the OARs are typically next to the PTV and the leaves only needed to move around the edge of PTV adjacent to OARs. Hence OARs could be spared well, even with low leaf motion. For hypolung cases, the target was located close to the periphery of the lung, and partial arcs with optimal beam angles were used to approach the tumor. The sparing of OAR was again not a problem (Fig. 2), even with a low leaf motion. Thus, by constraining leaf motion to lower speed, the algorithm tends to generate control points with larger segment shapes to deliver enough dose to the target, since the leaf motion range between adjacent control points has been effectively reduced. This, in turn, makes it easier to fulfill the dose objectives for the PTV. On the other hand, MUs go up quickly as the segment size is reduced. Hence, not constraining the leaf motion is not effective in reducing the total MUs and improving the target coverage.

#### A.2 Maximum delivery time

Maximum delivery time was thought to be a soft constraint but it had profound effect on the target coverage and was found out to be a very hard constraint. The final gantry speed for each control point is directly determined by the maximum delivery time specified by the user. An inverse relationship exists between the gantry speed and the maximum delivery time, which, in turn, can be estimated by dividing the arc length by the maximum delivery time. Longer maximum delivery time allowed the leaf to travel a wider range between the control points. A similar effect has been seen previously in leaf motion constraint settings. With the low maximum delivery time settings, the PTV coverage was comparable with the IMRT plan, but significantly improved with the increasing maximum delivery time. Hence, the maximum delivery time should be set as low as possible for low‐dose fractionation cases, like prostate and head‐and‐neck cases. For high‐dose fractionation cases, like hypolung and SRS spine, the maximum delivery time could be set higher to further improve the target coverage.

The dose rate distribution as a function of control points clearly shows the distribution to higher dose level and reduced MU fluctuation for both prostate and hypolung cases as the maximum delivery time decreased. The gantry speed increased due to a reduced maximum delivery time. In turn, the dose rate goes up to similar levels within the gantry angles. Higher dose rate distribution in dose rate enables less fluctuation in control point MUs because of shorter leaf travel, resulting in a smooth gantry rotation during delivery. For prostate, this effect was significant when the time was reduced from 90 s to 60 s. The effect was not noticeable beyond 90 s. This implies that if the maximum delivery time is set above 90 s, the algorithm will optimize a prostate plan without favoring the use of high dose rate values. In order to achieve a smooth delivery by utilizing high dose rate control points, the maximum delivery time needs to be set relatively low. This can be most efficiently achieved in hypolung cases, where the dose rate of all control points could be pushed above 550 MU/min by reducing the maximum delivery time to 180 s or lower.

#### A.3 Number of arcs and control point spacing

Typically, the delivery time and total MU goes up with increasing number of arcs and length of the arcs. The effect of using multiple arcs on MUs is much larger than the effect from increasing the maximum delivery time. The maximum delivery time had the largest effect on single arc in increasing total MUs. For two‐arc plans, SmartArc tends to distribute smaller segments into the second arc (Fig 4). So instead of using the dual arc option, the user should generate multiple arcs by creating separate beams for each of the individual arcs, which allows the user to configure the second arc different from the first arc to improve the segments distribution in the second arc.

Although dual arcs can provide better coverage, single and partial arcs were preferred in this study for cases where intrafraction motion could be significant (such as prostate and lung). It will be challenging to use single and partial arc to create a SmartArc plan for complicated cases (e.g., C‐shaped SRS spine cases). To make it feasible, the collimator angle can be set to 90 degrees for these single partial C‐shaped SRS spine plans. SmartArc is also not capable of generating multiple arcs from a single arc if the arc has a couch kick. The optimization results in a fatal error. The use of a third arc does not significantly increase the MUs, but to achieve similar PTV coverage, both MUs and estimated delivery time significantly goes up as the numbers of arcs is increased. Increasing the maximum delivery time is less efficient in terms of improving the PTV coverage of a multiple arc plan than that of a single arc plan.

In summary, we found that choosing larger degree control point setting has the same effect as constraining leaf speeds to lower values. Lower leaf speed reduces larger leaf travel between control points which, in turn, helps in reducing the total MU and delivery time. Since fewer number of control points are generated using larger control point spacing, SmartArc algorithm tends to use larger field size to provide adequate coverage to PTV with fewer control points, which again helps in reducing the total MU and delivery time. On the other hand, SmartArc plans with low control point spacing represent the actual delivery, as evident from the ArcCHECK QA results in Tables 1and 3. We believe choosing a 4 or 6 degree control point spacing may be adequate in achieving desired PTV coverage and smooth delivery using appropriate leaf speed and maximum delivery time.

## V. CONCLUSIONS

SmartArc is capable of generating efficient single and partial arc VMAT plans in terms of lower MUs, shorter treatment time, and better target coverage and OAR sparing compared to IMRT. Smoother segments are typically required for efficient delivery of plans and help in clinical situations, like lung SBRT, in order to minimize the interplay effect between the leaf motion and the patient motion. Our investigation on leaf speed and control point spacing will help the users in generating smoother and efficient SmartArc plans.

## ACKNOWLEDGMENTS

Authors would like to thank Kevin Reynolds from Philips for his help in initial planning using SmartArc and Derek Schulze for help with the interpolation script. Authors would also like to thank Nathan Berg for help in reviewing the article.
